# Drug Target to Alleviate Mitochondrial Dysfunctions in Alzheimer’s Disease: Recent Advances and Therapeutic Implications

**DOI:** 10.2174/1570159X22666240426091311

**Published:** 2024-04-27

**Authors:** Md. Ataur Rahman, MD. Hasanur Rahman, Hyewhon Rhim, Bonglee Kim

**Affiliations:** 1 Department of Pathology, College of Korean Medicine, Kyung Hee University, Hoegidong Dongdaemungu, Seoul, 02447, South Korea;; 2 Korean Medicine-Based Drug Repositioning Cancer Research Center, College of Korean Medicine, Kyung Hee University, Seoul, 02447, Korea;; 3 Global Biotechnology & Biomedical Research Network (GBBRN), Department of Biotechnology and Genetic Engineering, Faculty of Biological Sciences, Islamic University, Kushtia, 7003, Bangladesh;; 4 Center for Neuroscience, Brain Science Institute, Korea Institute of Science and Technology (KIST), 5 Hwarang-ro 14-gil, Seongbuk-gu, Seoul, 02792, Republic of Korea;; 5 Division of Bio-Medical Science and Technology, KIST School, Korea University of Science and Technology (UST), Seoul, 02792, Republic of Korea

**Keywords:** Alzheimer’s disease, mitochondria, mitochondrial dysfunction, drug target, therapeutic approaches, ROS

## Abstract

Alzheimer's disease (AD) is a severe progressive neurodegenerative condition associated with neuronal damage and reduced cognitive function that primarily affects the aged worldwide. While there is increasing evidence suggesting that mitochondrial dysfunction is one of the most significant factors contributing to AD, its accurate pathobiology remains unclear. Mitochondrial bioenergetics and homeostasis are impaired and defected during AD pathogenesis. However, the potential of mutations in nuclear or mitochondrial DNA encoding mitochondrial constituents to cause mitochondrial dysfunction has been considered since it is one of the intracellular processes commonly compromised in early AD stages. Additionally, electron transport chain dysfunction and mitochondrial pathological protein interactions are related to mitochondrial dysfunction in AD. Many mitochondrial parameters decline during aging, causing an imbalance in reactive oxygen species (ROS) production, leading to oxidative stress in age-related AD. Moreover, neuroinflammation is another potential causative factor in AD-associated mitochondrial dysfunction. While several treatments targeting mitochondrial dysfunction have undergone preclinical studies, few have been successful in clinical trials. Therefore, this review discusses the molecular mechanisms and different therapeutic approaches for correcting mitochondrial dysfunction in AD, which have the potential to advance the future development of novel drug-based AD interventions.

## 1 INTRODUCTION

Alzheimer’s disease (AD) is a progressive degenerative disease that impedes the cognitive and memory functions of the elderly worldwide [1]. Numerous studies have found that mitochondrial dysfunction may contribute to AD pathogenesis [2-4]. Important pathological traits in AD include amyloid β (Aβ) plaque accumulation, neurofibrillary tangle (NFT) formation by hyperphosphorylated tau protein (pTau), neuronal dystrophy, astrogliosis, biometal dyshomeostasis, increased oxidative stress, and decreased acetylcholine synthesis in the brain [5]. These phenomena have been associated with pre- and post-synaptic neuronal casualty. However, AD pathogenesis remains unknown, and no curative AD treatment has been found to date. Additionally, mitochondria are involved in key cellular processes, including calcium homeostasis, reactive oxidation species (ROS) production, apoptosis initiation, and metabolite secretion, controlling cell fate determination and function [6]. It has been reported that impaired mitochondrial function may significantly alter cellular and tissue homeostasis in AD. Furthermore, several studies have shown that genetic, biological, and environmental factors are associated with mitochondrial-mediated AD pathogenesis [7]. During AD development, mitochondrial function decreases, leading to changed mitochondrion morphology and synaptic space, reducing mitochondrial axonal transport levels [8]. Therefore, these deficits suggest that mitochondria dysfunction is associated with AD pathogenesis.

Neurodegenerative diseases are chronic debilitating conditions defined by the progressive and selective degeneration of neurons in the central or peripheral nervous system. Primary age-related neurodegenerative illnesses, such as AD and Parkinson's disease (PD), result in dementia, a leading cause of impairment globally and a significant public health burden with escalating healthcare costs. Mitochondrial dysfunction is associated with decreased energy production, poor calcium buffering, protease and phospholipase activation, and increased oxidative stress in AD and PD. Increased oxidative stress and accumulation of mitochondrial DNA (mtDNA) mutations result in mitochondrial malfunction and play a crucial role in the aging process and AD and PD development. AD is the most common cause of dementia. It is marked by the extracellular buildup of Aβ peptides in senile plaques and the intracellular buildup of tau protein in NFTs in the brain’s gray matter. When Aβ and pTau aggregates interact, metabolic decline occurs in limbic structures, such as the cingulate, orbitofrontal, medial, and basal temporal cortices, leading to AD.

Changes in the size and number of mitochondria in neurons are indicative of potential changes in mitochondrial dynamics in AD. However, the presence of proteinaceous deposits within neuronal perikarya (Lewy bodies) and processes (Lewy neurites) is one of the pathological hallmarks of sporadic PD. These deposits primarily comprise alpha-synuclein, ubiquitin (UBB), neurofilaments, and molecular chaperones. What function Lewy bodies play in PD progression and whether they are a pathognomonic sign of PD remains unclear. Rotenone is a well-known inhibitor of complex I of the mitochondrial electron transport chain (ETC) that has been used to induce mitochondrial dysfunction and as a preclinical PD model. It inhibits mitochondrial complex I causing loss of nigrostriatal neurons, rigidity, hypokinesia, and the buildup of alpha-synuclein- and UBB-containing fibrillar inclusions. Moreover, it causes neuronal death *via* oxidative stress, adenosine triphosphate (ATP) depletion, endoplasmic reticulum stress, and the expression of activating transcription factor 4 (*ATF4*), phosphorylated pancreatic endoplasmic reticulum kinase/PKR-like endoplasmic reticulum kinase (*PERK*), immunoglobulin heavy-chain binding protein (*HSPA5*), and DNA damage-inducible protein/C/EBP homologous protein (*CHOP*).

There is increasing evidence indicating that mitochondrial dysfunction occurs in healthy aging and diseases, particularly AD [5]. Mitochondrial dysfunction in AD induces phosphorylation of tau and Aβ accumulation. In addition, mitochondria contribute to various biochemical pathways in cells, including steroid hormone synthesis, calcium homeostasis, ATP production, apoptosis, and energy efficiency control [9]. Currently, it is well-known that central cellular pathways are compromised in AD with intraneuronal NFTs made of pTau and extraneuronal senile plaques (SP) made of Aβ, vascular damage, synaptic failure, neuronal and axonal injury, oxidative stress, and microglia-mediated neuroinflammation [10]. Therefore, current efforts are directed toward exploring the potential targets and underlying mechanisms of mitochondrial dysfunction in AD pathogenesis, focusing on describing the function of mitochondrial dysfunction and the status of mitochondrial therapy in AD.

## 2 MOLECULAR MECHANISM OF AD

**Fig. (1):**
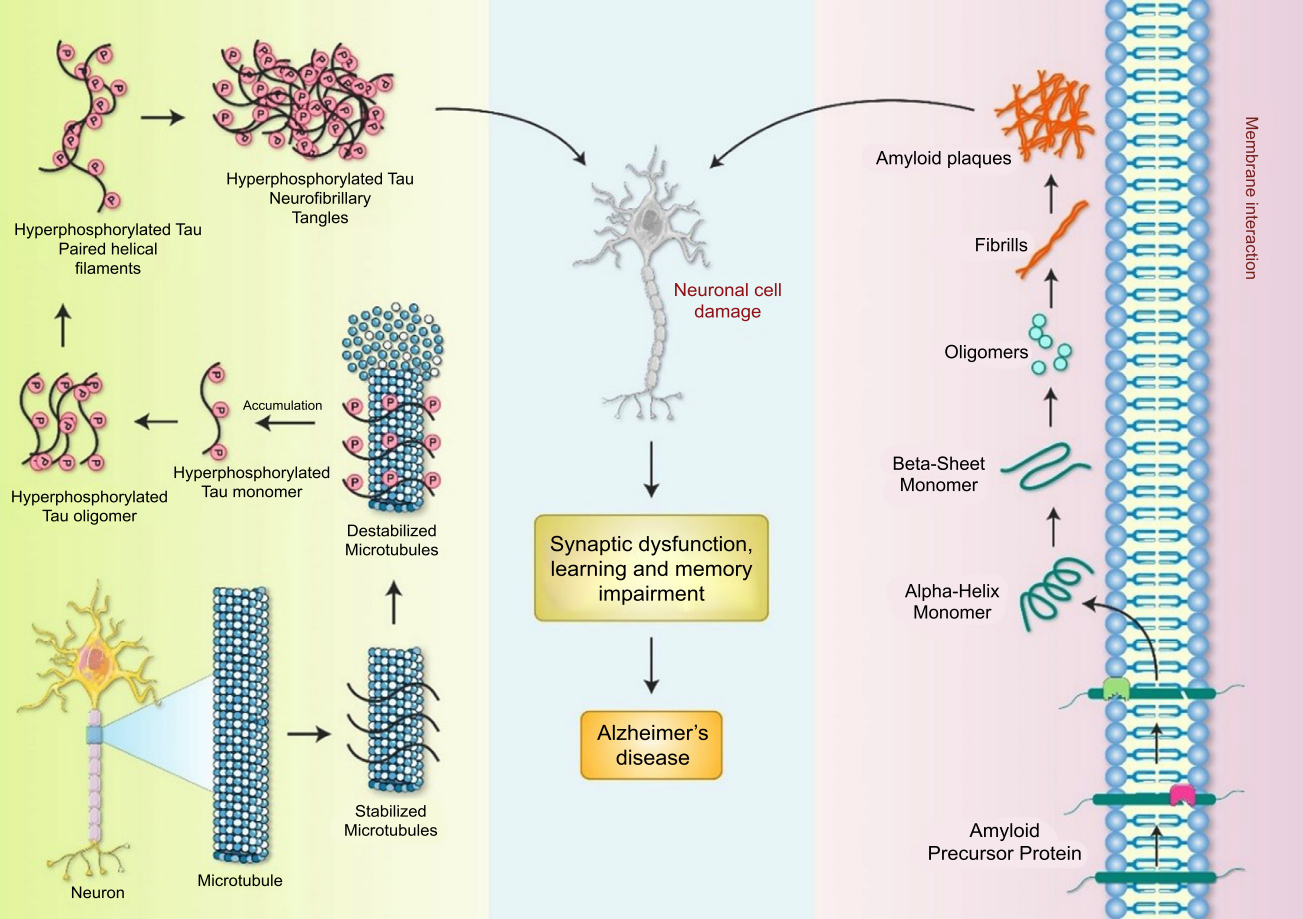
The molecular pathophysiology of AD. APP is cleaved by α-secretases and β-secretases, resulting in the accumulation of neurotoxic Aβ in plaques. NFTs are aggregates of pTau protein in the nervous system. AD is characterized by synaptic disruption and memory impairment caused by Aβ plaques and NFTs.

AD is a common neurodegenerative disorder characterized by memory loss and the development of pTau and Aβ aggregates in numerous brain areas, including the hippocampus and cortex [11]. The clinical and pathological AD manifestations include memory impairment and sluggishness when performing typical daily tasks [12]. The principal pathogenic hallmarks are intracellular aggregates formed by extracellular Aβ and pTau protein deposition, creating NFTs and contributing to SP development [13-16] (Fig. **1**). Tau protein aggregates have been found in the proximal axon, and large, insoluble tau aggregates containing irreversibly pTau species do not migrate through axons [13]. Toxic tau species may harm the transport mechanism in the axon, reducing amyloid precursor protein (APP) transport to the synapse, thus causing APP buildup in the soma and Aβ aggregation [17]. The hyperphosphorylation of the microtubule-associated protein tau causes it to aggregate in an insoluble state, creating NFTs [18]. The NFT development process and whether NFTs are a major cause of illness or play a more peripheral function remains unknown. The existence of NFTs has been found to be strongly associated with the degree of cognitive impairment in disorders, such as AD [19]. Investigating potential associations between tau and NFTs is crucial for gaining a mechanistic understanding of events contributing to the onset of AD [19]. Mitochondrial dynamics are disrupted in AD, leading to the identification of fission protein inhibitors, such as dynamin-related protein 1 (DRP1) and drugs that induce fusion [20]. Modifications to the adenosine monophosphate (AMP)-activated protein kinase (AMPK), sirtuin 1 (SIRT1), and protein kinase B (AKT) pathways may potentially be superior therapeutic options because these pathways influence mitochondrial activities [21]. Oxidative phosphorylation is a primary source of ROS, which causes mitochondrial damage in AD [22].

## 3 FUNCTIONAL ROLE OF MITOCHONDRIA

Mitochondria are the cell's energy producers, supplying the majority of ATP *via* oxidative phosphorylation [23]. Mitochondria are comprised of compact sac-like structures made up of two layers, the outer (OMM) and inner (IMM) mitochondrial membranes, which are chemically and structurally different [6]. The OMM is composed of equal parts lipids and proteins and can be transited by molecules with a molecular weight of 6 kDa [24]. IMM is a highly specialized unit membrane that folds inward to produce a ridge, increasing its surface area [25]. IMM has poor permeability, and proteins make up ~76% of its total weight. Many chemicals, such as hydrogen ions (H^+^), ATP, and pyruvate, require carriers to transport them across the IMM [26]. The intermembranous gap is located between the OMM and the IMM. The mitochondrial matrix encompassed by the IMM contains different enzymes, ribosomes, and mtDNA and RNA [27]. These traits might be exploited to develop mitochondrial-targeted strategies in AD pathology. A detailed mitochondrial schematic representation is shown in Fig. (**2**).

Neurons consume the most ATP of any cell type to sustain the ionic gradients required for continuous neurotransmission, electrophysiological activity, and short-term synaptic plasticity [28]. The primary purpose of mitochondria is to provide ATP and oxygen to the cell, which requires electron transport *via* the ETC [29]. Located on the IMM, the ETC comprises five proteins, complexes I-V. Complexes I, III, and IV function as proton pumps that transfer H^+^ from the intermembranous region to the matrix [30]. Consequently, a proton gradient is created, resulting in a significant negative internal membrane potential (ΔΨm) on the IMM of roughly 160 to 180 mV, influencing the entrance of drugs into the mitochondria [31]. Surprisingly, around 0.4-4% of the electrons traveling through the ETC are not fully restored, resulting in the production of primary ROS-superoxide anions (O^2-^). Excessive superoxide anion production interacts with various different substances to produce secondary ROS and cause oxidative damage [32]. Many ROS by-products are produced in the brain due to high energy demand and rapid ATP production/consumption. ROS overproduction or dysregulation of the antioxidant system disrupts normal mitochondrial activity, resulting in inflammation, apoptosis, memory loss, and brain/neuronal damage [33]. Mitochondrial dysfunction is key in the etiology of AD [34]. Neuronal mitochondrial dysfunction caused by chronic ROS production accelerates the degenerative process underlying AD, such as increased Aβ aggregation and NFT formation [35].

## 4 MITOCHONDRIAL DYSFUNCTION IN AD PATHOGENESIS

It has been found that mitochondrial functions and cellular homeostasis are sustained by the dedicated mitochondrial quality control system (mtQCS) [1] that contains diverse biochemical mechanisms that act at different levels, from individual polypeptides to the whole organelle [1]. However, the morphology and role of mitochondria are strictly associated with continuous fusion-fission dynamics in the cellular system [36]. The cause and effects of mitochondrial dysfunction in AD pathogenesis are illustrated in Fig. (**3**). While evidence of mitochondrial dysfunction in AD has been discussed, the actual cause remains unknown, and the defective metabolism characteristic of AD may be simply reflecting mitochondrial dysfunction.

### 4.1 Dysfunction of Amyloid Cascade in AD

Over the last 30 years, the amyloid cascade hypothesis has dominated AD research and is supported by evidence. Firstly, extraneuronal SPs are comprised of Aβ peptides, and mutations in its precursor APP cause an early-onset form of AD [37,38]. Secondly, Aβ-induced mitochondrial fission is increased in the presence of S-nitrosylated DRP1 [39]. Furthermore, Aβ plaque accumulation causes cellular mitochondrial dysfunction toxicity and abnormal mitochondrial structure [17]. Conversely, dysfunctional mitochondrial morphology in AD neurons inhibits mitochondrial function and reduces mitochondrial content in addition to neuronal function [40]. However, this hypothesis has lost importance following the failure of Phase III clinical AD trials and must be revised or incorporated into other hypotheses [41].

### 4.2 Dysfunctional Mitochondrial Dynamics in AD

Recently, it has been shown that mitochondrial morphology and function are intimately associated with the continuous fusion-fission dynamics in cells [42]. The five essential proteins involved in controlling dynamics are mitochondrial fission protein 1 (FIS1), DRP1, optic atrophy 1 (OPA1), and mitofusin 1 (MFN1) and 2 (MFN2) [43]. Mitochondria fusion and fission proteins are variably present in the AD hippocampus, with increased *FIS1* and significantly decreased *DRP1* expression alone or with the fusion proteins *OPA1*, *MFN1*, and *MFN2* [44]. During cell division, the fusion-fission cycle controls genetic complementation, mitochondrial functionalization, and the correct distribution of newly generated mitochondria [45]. Conversely, in AD, more frequent fission than fusion may increase the overall quantity of palingenetic mitochondria. Similarly, *APP* overexpression in neural cells was found to cause mitochondrial fragmentation by modifying levels of mitochondrial fusion and fission proteins [46].

### 4.3 Dysfunctional Mitochondrial Biogenesis in AD

Mitochondrial biogenesis generally occurs regularly in normal cells and in response to oxidative stress and increased energy consumption in some neurodegenerative disorders [47]. Mitochondrial biogenesis compensates for damaged mitochondria and is critical in sustaining an appropriate functional mass of neuronal mitochondria in AD [48]. Coordination and interaction between the nuclear (nDNA) and mtDNA play a significant role in this process during AD. Additionally, recent studies have shown that proliferator-activated receptor coactivator 1 (PGC-1) can control mitochondrial biogenesis by activating transcription factors, including nuclear respiratory factors 1 (NRF1) and 2 (NRF2) and mitochondrial transcription factors A (TFAM) [49]. Similarly, PGC-1 activity is influenced by mitochondrial damage, nutrient availability, and energy balance in cells [50]. Damaged mitochondria were associated with lower NRF1, NRF2, TFAM, and PGC-1 levels in the HEK293-APPswe AD cell model [51]. Furthermore, other studies have found decreased PGC-1-mediated mitochondrial biogenesis signaling in mice with early-stage AD [52]. Therefore, reduced mitochondrial biogenesis is a significant feature of AD.

### 4.4 Dysfunction of Mitochondrial Membrane Potential in AD

A mitochondrial membrane potential (MMP) is generated when protons are pumped from the mitochondrial matrix into the intermembrane space *via* the ETC [53]. MMP is the most important component of the mitochondrial electrochemical potential gradient, the depletion of which leads the ETC to uncouple ATP phosphorylation [54]. Therefore, lowering local oxygen tensions by restricting the half-life of ETC intermediates results in a modest drop in MMP and reduced ROS production [55]. However, studies have shown that APP/PS1 transgenic mouse brains have lower ATP levels and complex IV activity and greater oxidative stress than controls [56]. Furthermore, MMP levels are lower in AD animal models and human cortical neurons.

### 4.5 Dysfunctional Ca^2+^ Homeostasis in AD

The high-capacity Ca^2+^ pool decreases with normal neural activity, and mitochondria play an important role in preserving cellular Ca^2+^ homeostasis by keeping mitochondrial Ca^2+^ levels aligned with variations in cytosolic Ca^2+^ load [57]. Mitochondria use an ATP-powered or MMP-driven Ca^2+^/H^+^ pump to take up Ca^2+^ in association with H^+^ [58]. Furthermore, the opening of Ca^2+^-mediated permeability holes is functionally connected to another Ca^2+^ absorption pathway [59]. Excess Ca^2+^ absorption *via* mitochondria promotes ROS production, slows ATP synthesis, activates the mitochondrial permeability transition pore (mPTP), and sometimes even causes cell death [60]. Accordingly, Aβ buildup can cause cellular damage in neurons by promoting ROS production and altering Ca^2+^ homeostasis in mitochondria [61].

### 4.6 Dysfunctional Mitophagy in AD

Mitophagy is the process by which injured mitochondria are enveloped by autophagosomes and transported to lysosomes for degradation and recycling [62]. Impaired autophagy has been found to cause a buildup of aberrant mitochondria in AD neuronal cells, exacerbating mitochondrial dysfunction [63]. While mitophagy is induced by ROS produced by injured mitochondria, excessive ROS production suppresses this process [64]. Interestingly, the mammalian target of rapamycin (mTOR) complex, which is highly active in the AD hippocampus brains, directly regulates mitophagy [64]. Recently, mitophagy was found to reduce Aβ and tau pathology and cognitive impairment in AD pathogenesis [65]. Nevertheless, impediments to the clearance of damaged mitochondria and cellular oxidative stress might cause the aggregation of defective AD brain neurons [66]. Consequently, developing drugs that target mitochondrial malfunction to restore MMP and Ca^2+^ equilibrium might provide a new paradigm for AD treatment. Therefore, if mitophagy is also substantially impaired in AD, it could lead to the buildup of damaged mitochondria and malfunctioning neurons, potentially disrupting the fusion of autophagosomes and lysosomes in AD.

## 5 EMERGING TREATMENT APPROACHES TO TARGET MITOCHONDRIAL DYSFUNCTION IN AD

A viable and practical method for slowing or reducing brain damage is the design of pharmaceutical molecules that target mitochondrial dysfunction to modify mitochondrial bioenergetics for homeostasis by restoring mitochondrial function. Currently, the standard treatments for AD are cholinesterase inhibitors (donepezil, galantamine, and rivastigmine) and memantine, which inhibit the N-methyl-D-aspartate (NMDA) receptor and excess glutamate activities [67]. NMDA receptors and acetylcholine (ACh) are essential in memory and learning functions, and their concentration and function are impaired in AD [68]. However, these drugs improve cognitive and memory function without slowing disease development [69]. Moreover, since the concept of AD as a multifaceted illness has gained traction recently, a reassessment of mitochondrial-targeted therapy in combination with other drugs is strongly advised. Currently, the US Food and Drug Administration (FDA) has approved four drugs to treat AD. Acetylcholine esterase (AChE) inhibitors are a group of three drugs: donepezil, rivastigmine, and galantamine. Memantine, which blocks NMDA receptors, is the fourth treatment option, and combined donepezil and memantine is the fifth [70]. Recent AD studies have focused on the gut and its inhabitants, the microbiome, which represents another important therapeutic consideration [70].

### 5.1 Phytochemicals Targeting Mitochondrial Dysfunction in AD

**Fig. (4):**
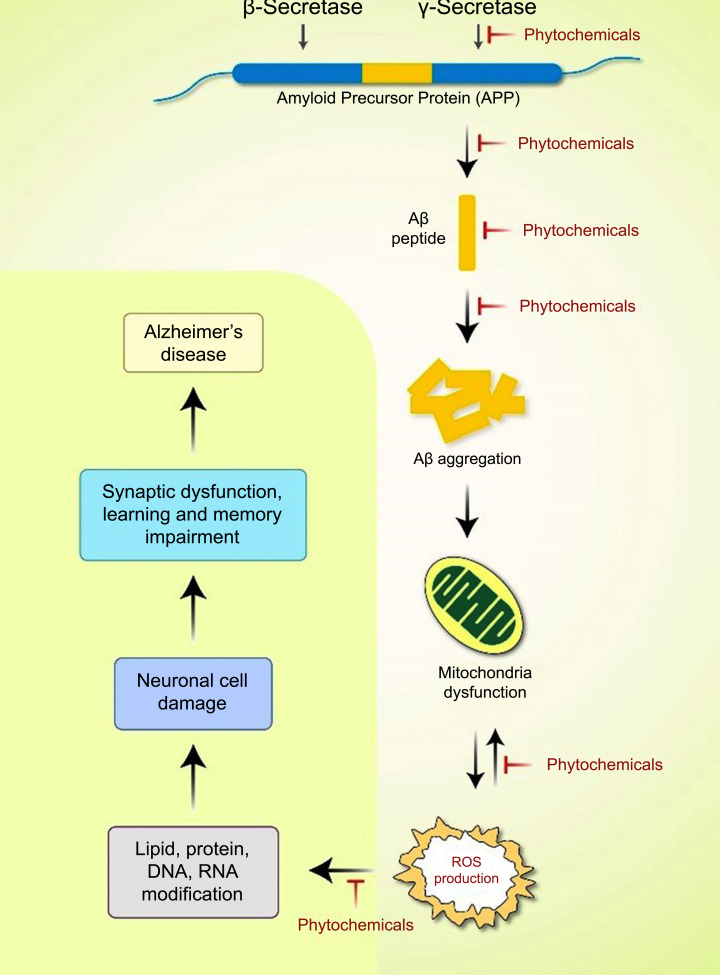
Phytochemicals are emerging as potential treatments for mitochondrial dysfunction in AD development. Abnormal APP is digested by the β- and γ-secretases, resulting in the buildup of extracellular Aβ. When there is insufficient clearance of Aβ or Aβ production, aggregation occurs, resulting in the buildup of diverse Aβ assembly types. Aβ accumulation is directly associated with mitochondria, and ROS production is directly associated with other intracellular pathways. Neurological degeneration and synaptic function dysregulation in brain regions implicated in learning and memory impairment in AD are caused by these oxidative stress reactions, which have a multifactorial mechanism of action, in addition to neurological degeneration and synaptic dysregulation function.

Several studies have shown the beneficial treatment potential of antioxidants and mitochondria-targeting drugs, such as vitamin C, vitamin E, carnitine, and alpha-lipoic acid in AD [71]. Coenzyme Q10, curcumin, piracetam, simvastatin, piracetam, ginkgo biloba, and omega-3 polyunsaturated fatty acids have also been effective therapeutic agents [72]. A viable treatment method for AD that targets mitochondrial proteins can be developed, and various mitochondria-targeted antioxidants have been created with this approach. Changes in mitochondrial mobility have a deleterious effect on mitochondrial function, contributing significantly to AD development [73]. Consequently, efforts to correct faulty mitochondrial mobility and transport may constitute a viable therapeutic strategy for AD treatment. Therapeutics that inhibit the activation of mitochondrial fission proteins, such as DRP1, pTau, and Aβ, can protect neurons against the harmful effects of these drugs and their interaction. A wide range of phytochemicals found in various plant sources show various pharmacological effects, including apoptosis induction [74-81], neuroprotection [82, 83], autophagy activation [84-88], antioxidant effects [89], DNA repair functions [90], and anti-inflammatory activity [91].Therefore, phytochemicals are increasingly evaluated as promising therapeutic possibilities for AD treatment because of these properties [92] (Fig. **4**).

Melatonin is a natural compound derived from plants and animals that increases mitochondrial biogenesis factors, such as PGC-1, NRF1, NRF2, and TFAM, MMPs, such as Na^+^-K^+^-ATPase and cytochrome C (Cyt-c), ATP levels, mtDNA/nDNA ratio, and mitochondrial structure, and decrease amyloidogenic APP processing in AD [51]. Melatonin has a strong neuroprotective effect and can stop or slow AD progression, supporting the view that it could be used to treat AD. Melatonin alters the transcription regulatory network and activity of secretases, inhibiting amyloidogenic APP processing and Aβ production [93]. Therefore, further research is required to investigate the modulating effects of melatonin on the structure and function of mitochondria in AD.

Curcumin's neuroprotective impact on AD is widely recognized. Curcumin protects SH-SY5Y human neuroblastoma cells against Aβ-mediated mitochondrial dysfunction and synaptic damage [94]. In preclinical trials, quercetin restored mitochondrial dysfunction by restoring MMP, resulting in decreased ROS production and restoring ATP synthesis [95]. Moreover, this therapy dramatically increased *AMPK* expression, reduced dispersed senile plaque formation, and suppressed learning and memory impairment [95]. Long-term oral quercetin treatment to triple transgenic AD mice resulted in decreased tauopathy, astrogliosis, microgliosis, and amyloidosis in the amygdala and hippocampus, which enhanced cognitive function, learning performance, and spatial memory function [96, 97].

Different phytochemicals and other chemicals used in mitochondrial-targeted AD treatments in preclinical and clinical studies are listed in Table **1**. Human trials are being conducted to determine whether omega-3 fatty acids obtained by eating fish can prevent coronary artery disease, stroke, aging, dementia, and AD [98]. Conversely, flavonoids and polyphenols from Mediterranean diets have antioxidant and anti-inflammatory effects on cardiovascular disease, type 2 diabetes, cancer prevention, and stroke in humans [98]. Polyphenols found in fruits and vegetables have been shown to control tau hyperphosphorylation and Aβ aggregation in animal AD models [98]. The soy isoflavonoid genistein has therapeutic potential in various aging-related mitochondrial dysfunction in pathological conditions, such as neuroinflammation, oxidative stress, and Aβ aggregation in AD [99]. Its therapeutic effect was related to its capacity to ameliorate mitochondrial function deficits caused by Aβ aggregates [100]. A larger dosage of genistein (150 mg/kg/day) was recently shown to activate autophagy in the streptozotocin-induced rat model of sporadic AD [101]. Furthermore, therapy with genistein resulted in the total degradation of tau hyperphosphorylation and Aβ protein in brains with mitochondrial dysfunction. Recently, genistein-loaded nanocomposites have been developed, showing the potential of oral delivery and overcoming the harmful isoflavonoid effects [102].

### 5.2 Mitophagy-targeted Mitochondrial Dysfunction in AD

The efficient removal of old and malfunctioning mitochondria *via* mitophagy, a cargo-selective form of autophagy, is important for the preservation of mitochondrial function and neuronal health [113]. Mitophagy has been the subject of numerous mechanistic studies, which have uncovered a complex and interconnected cellular network governing mitochondrial turnover [114]. Impaired mitophagy occurs early in AD brains and plays a causal role in the development of AD-associated neuropathology. The findings of various AD studies suggest that increased mTOR activity may result in deficient mitophagy in the hippocampus and other brain locations [55]. More importantly, mTOR phosphorylation levels are altered in the brains of AD patients and are associated with the presence of pTau [115]. Consequently, modulation of the mTOR signaling pathways may prove to be an effective strategy for AD intervention and treatment. The mTOR inhibitor rapamycin was shown to be effective in AD treatment, restoring mitophagy/autophagy, increasing the levels of microtubule-associated protein 1 light chain 3 (LC3) II/I and other autophagy-related proteins, suppressing the mTOR pathway [16, 116, 117]. Consequently, mTOR inhibition has garnered much attention as a potential AD treatment. Pharmacological reinstallation of mitophagy has beneficial effects on amyloid and tau pathologies in AD animal models with therapeutic effects on memory loss [118]. Further studies using neurons grown from induced pluripotent stem cells (iPSCs) of sporadic AD or other comparable models could be extremely important in determining whether mitophagy failure is a crucial factor in the development of Aβ/tau proteinopathies.

**Fig. (5):**
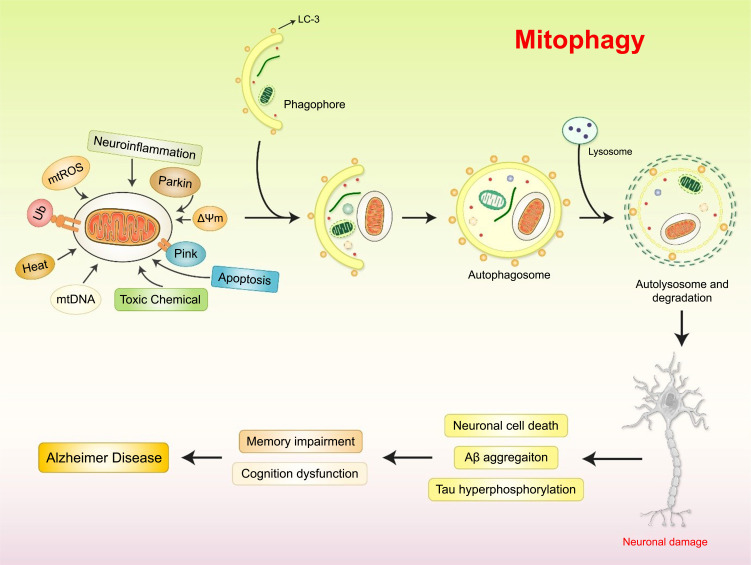
Mitophagy mechanism in AD pathogenesis. Parkin ubiquitylates various OMM elements, which are then identified by the adaptor proteins optineurin (OPTN), p62, nuclear dot protein 52 kDa (NDP52), and NBR1 autophagy cargo receptor (NBR1), recruiting the damaged mitochondria to the autophagy process and triggering autophagosome production *via* interactions with LC3. Additionally, MMP, toxic chemicals, mitochondrial ROS (mtROS), heat, and neuroinflammation are keys factor in initiating mitochondrial-mediated autophagy, damaging neuronal cells.

Mitophagy involves identifying malfunctioning or redundant mitochondria, developing and maturing phagophores, fusion with the lysosome, and degrading mitochondria (Fig. **5**). Reduced MMP promotes the stability of PTEN-induced kinase 1 (PINK1) in the OMM. PINK1 is activated by autophosphorylation, after which it phosphorylates MFN2 and UBB, resulting in the recruitment of Parkin to the OMM surface [119]. A basic understanding of mitophagy deficiency in AD progression and the fundamental molecular mechanisms that regulate mitophagy are required before understanding the interplay between AD pathology and mitophagy deficiencies.

Latrepirdine is an antihistamine drug that reduces mitochondrial enlargement under Aβ stress in cell cultures of AD mice models and stabilized MMPs [120]. The collaboration between latrepirdine and glutamate receptors decreased mitochondrial permeability and blocked voltage-dependent calcium channels in HEKsw cells, preventing unwanted mitophagy or apoptosis [120]. In addition, there is accumulating evidence that EGb761 extract suppressed the mPTP production and reduced tau hyperphosphorylation and cognitive impairment in AD rat models [121]. Clinical trials examining the efficiency of EGb761 extract in treating dementia have indicated that it has beneficial effects on cognition and daily activities [122]. Consequently, mPTP inhibition in mitophagy is a promising therapeutic target for AD treatment.

Inducing mitophagy *via* pharmaceutical treatment reduced neuroinflammation and improved cognitive performance in an AD mouse model. Increased expression and activity of the NLR family pyrin domain containing 3 (NLRP3) inflammasome have been found in the brain tissues of APP/PS1 AD mice [123]. However, restoration of neuronal mitophagy with the mitophagy-inducing chemicals urolithin A and actinonin reduced neuroinflammation, indicated by decreased levels of cleaved caspase 1 (CASP1), proinflammatory interleukin 1β (IL-1β), and IL-1β [124]. Furthermore, it may be beneficial to design drugs or techniques to activate the NAD^+^‐dependent protein deacetylase SIRT1 to protect against AD [125]. Photobiomodulation therapy (PBMT) activated the cyclic AMP (cAMP)/protein kinase A (PKA) pathway, increasing SIRT1 deacetylase activity in APP/PS1 AD neurons [120]. Therefore, AD could be treated by PBMT-mediated *SIRT1* overexpression, thus reducing Aβ production [126], indicating that targeting mitophagy pathways may have potential therapeutic effects on AD.

### 5.3 Lifestyle Modification and Physical Activity in AD

A Mediterranean diet, calorie restriction, and physical activity can improve human aging and decrease the risk of neurological diseases [127]. The Mediterranean diet is comprised mostly of fruits, vegetables, and omega-3 fatty acids, which are abundant in olive oil and fish. Studies have found that extra-virgin oil rich in polyphenols decreased mitochondrial-associated oxidative stress and insulin resistance in rats fed a high-fat diet [128]. In addition, oleuropein aglycone (OLE), another polyphenol ingredient of olive oil, stimulated autophagy, decreased aggregated protein levels, and decreased cognitive impairment in AD patients’ brains [129]. Another bioactive component in olive oil, hydroxytyrosol (HT), improved mitochondrial dysfunction in an AD animal model [130].

When there is a limited supply of glucose available in the brain, ketones can be used as an alternative energy source. A ketone ester diet had beneficial effects on mitochondrial function in the 3xTg AD model [127]. Therapeutic ketosis has been proposed to prevent AD brain pathology progression, including the buildup of plaques and NFT [131]. Additionally, calorie restriction (CR), reduced calorie consumption without nutrient deficiency, is a promising approach to enhance longevity and insulin sensitivity and prevent age-related diseases [132]. CR increased mitophagy and ATP levels, decreased ROS, and improved mitochondrial quality and cell bioenergetics in AD [133]. Positive effects of CR were observed at the mitochondrial level, where it was found to influence mitochondrial biogenesis *via* activation of NO synthase (eNOS) [134].

Physical exercise (PE) has a positive effect on physical and mental health, including brain plasticity and cognitive function. PE treatment improved mitochondrial respiratory function by increasing respiratory chain complex (RCC) activity and reducing ROS production capacity in conjunction with Aβ_1-42_ peptide levels and improved cognitive function in the hippocampus of the APP/PS1 transgenic AD mouse model [135]. Maternal exercise while pregnant had a beneficial effect on mitochondrial function at the onset of AD. Moreover, it also had a preventive role against Aβ oligomer-induced neurotoxicity in the brains of adult offspring rats [136]. Clinical PE trials were conducted in older individuals with healthy or impaired cognitive function, leading to altered Aβ_1-42_ levels in plasma and cerebrospinal fluid (CSF). Altered cognitive and executive functioning, hippocampus volume, and memory were observed and were associated with reduced brain atrophy [137, 138]. Therefore, all animal-model-based research supports the view that PE may also have beneficial effects on mitochondrial function and glucose metabolism in humans.

### 5.4 Glucose Metabolism Targeting Mitochondrial Dysfunction in AD

In different neurodegeneration models, such as AD, mitochondrial dysfunction, decreased glucose uptake, and diminished glucose metabolism have been observed. During the early stages of AD, impaired insulin signaling, caused by decreased glucose consumption and inefficient energy metabolism, is a critical AD indicator [139]. A decreased insulin response is associated with defective mitochondrial glucose consumption in the 3xTg AD mouse model [140]. Insulin treatment in the hippocampus can improve Aβ-induced memory dysfunction by activating major hippocampal extracellular signal-regulated (ERK) and mitogen-activated protein (p38) kinases [120, 141]. The effects of that long-acting insulin medication detemir, delivered intranasally for three weeks, have been examined in individuals with AD or amnestic moderate cognitive impairment [142]. Consequently, efforts to alter the regulatory link between insulin signaling and glucose metabolism in mitochondrial dysfunction are promising options for memory restoration and AD treatment.

### 5.5 ETC Targeting in Mitochondrial Dysfunction in AD

Candidate ETC-targeting compounds have been explored as potential AD treatments. The disruption of ETC function caused by Aβ accelerated tau phosphorylation, and polymerization caused NFT formation in AD [143]. Neurotrophic treatment agents, such as the curcumin derivative J147, have a beneficial effect in preventing or reducing AD progression [144]. Additionally, the J147 treatment improved memory and cognition in APP/PS1 mice while still maintaining synaptic protein levels [145]. AMPK/mTOR pathway modulation by J147 protected against age-related brain toxicities by directing ATP synthase and modifying the mTOR pathway [145]. Consequently, using agents and drugs that target the ETC in dysfunctional mitochondria is a potential treatment approach for AD.

### 5.6 Nanoparticle-based Treatment of Mitochondrial Dysfunction in AD

Currently, targeted AD drug delivery to the central nervous system (CNS) is hindered by the difficulties posed by the blood-brain barrier (BBB) surrounding the CNS, limiting the bioavailability of therapeutic agents [146]. Nanoparticles (NPs) are a promising new strategy being developed to overcome these limitations and successfully deliver drugs to the CNS, providing new therapeutic options for efficiently transporting drugs across the BBB and into the brain [146]. The most common NP forms used in developing innovative AD therapeutics are illustrated in Fig. (**6**). Additional research is needed to identify the most effective NP-based therapeutics for mitochondrial dysfunction in AD.

At present, nanotechnology is a potential technique for delivering therapeutic AD drugs into mitochondria. However, NPs created using inorganic materials have several limitations. Nanostructured lipid carriers (NLC) carrying rabies virus glycoprotein RVG29 and triphenylphosphine (TPP) molecules attached to the red blood cell (RBC) membrane surface (RVG/TPP NPs@RBCm) and were effective in targeting neurons and localizing to their mitochondria [147]. In addition, RVG/TPP-respiratory syncytial virus (RSV) NPs@RBCm were effective in alleviating AD symptoms by reducing Aβ-related mitochondrial oxidative stress in cell and animal models [148].

Several antioxidants, including lipoic acid, glutathione, vitamin C, and vitamin E, have proven effective in clinical studies on moderate cognitive impairment and AD [149, 150]. In addition, benzoquinone idebenone effectively inhibited Aβ-induced neurotoxicity *in vitro* and *in vivo* by targeting mitochondria [133]. Notably, biodegradable polylactic-co-glycolic acid (PLGA) NPs exert neuroprotective effects in AD treatment [87], and coenzyme Q10 (CoQ10)-loaded PLGA NPs protected against Aβ cytotoxicity and restored memory in an AD mouse model [151].

Inorganic monomer, gold carrier, magnetic core, and carbon-based NPs with graphene oxide (GO) sheets have been used to remove Aβ aggregates and inhibit Aβ fibrillation [152]. The most effective NPs for disrupting Aβ aggregation with low cytotoxicity *in vitro* were GO/gold nanoparticles. In addition, nano-metallo-supramolecular complexes effectively inhibited Aβ-induced biosynthesis of heme and iron uptake by PC12 cells [153]. Liposomes (LIP) biofunctionalized with murine apolipoprotein E (mApoE) and phosphatidic acid (PA), a high-affinity ligand for Aβ, to facilitate the bridging of the BBB decreased Aβ plaque load, which might be beneficial in AD treatment [154]. Recently, AD treatment has been developed based on solid lipid nanoparticles (SLNs) functionalized with anti-transferrin receptor monoclonal antibody OX26 acting as the carrier and successfully transporting a bioactive extract across an *in-vitro* human BBB model [155]. Some nanotechnology-based approaches are summarized in Table **2**.

### 5.7 Additional Mitochondria-based AD Therapies

*In vitro* and *in vivo* studies on AD models have shown that nicotinamide adenine dinucleotide (NAD) therapies have a direct and favorable effect on mitochondrial function. Surprisingly, after 6 months of treatment, the participants with likely AD showed no cognitive decline, indicating that NAD may represent a promising strategy for preventing AD progression [166]. The antihistamine Dimebon used to treat allergies was examined by an AD clinical study due to the improved cognition and memory of neurotoxic rats treated with it [167].

Pioglitazone is a common global AD preventive treatment that slows AD development from mild cognitive impairment (MCI) depending on their APOE and translocase of OMM 40 (TOMM40) genotypes and Aβ status [168]. The Krebs cycle and gluconeogenesis intermediate oxaloacetate have already been proposed as a novel AD treatment and studied in AD patients [169]. Mouse-based oxaloacetate studies found beneficial effects on glycolysis, respiratory fluxes, mtDNA, mtDNA-encoded proteins, mitochondrial biogenesis, neuroinflammation, hippocampus neurogenesis activity, and altered brain insulin signaling [170].

## 6 LIMITATIONS AND FUTURE DIRECTIONS OF MITOCHONDRIAL DYSFUNCTION IN AD

Currently, no approved AD-modifying treatments can remove these proteins from AD patients' brains. Mitochondrial function is very important in AD, and correcting mitochondrial deficiency has emerged as an attractive potential approach for preventing or halting AD. Current preventative therapies for mitochondrial dysfunction are mostly focused on anti-apoptotic medicines, antioxidants, and natural agents that improve glucose metabolism and mitochondrial bioenergetics. Numerous therapies have shown clear cognitive benefits in preclinical studies on AD mouse models. Several ongoing studies are seeking new and potentially effective AD drugs. However, most therapies have not been completely successful in preventing, postponing, or retreating cognitive decline in clinical studies. Moreover, there is a general lack of effective cell and animal models that simulate the general pathophysiological circumstances and intricate etiology of AD. In addition, various limitations have been found for these candidates, impeding their use in AD treatment, including poor BBB diffusion, low bioavailability, and a restricted capacity to sustain the half-life of dose-response actions. Consequently, great efforts have recently been made to overcome these limitations by attaching candidates and their derivatives to liposomes, lipids, micelles, metal complexes, and NPs.

Further broad studies on mitochondrial activity are required to discover abnormalities shared by most AD patients since few previous studies have examined mitochondrial function in AD with large patient cohorts. Future methods should target early antecedent impairments in substrate supply apoptotic pathways and mitochondrial functions in bioenergetic systems to avoid the development of permanent AD pathology. Mitochondria play important roles in neurons in maintaining appropriate neural synapses, signal transmission, and other critical neuronal activities, indicating that addressing mitochondria dysfunction may be a promising therapeutic strategy for AD treatment. The failures of previous pharmaceuticals in clinical trials often occurred because the underlying scientific basis was not always robust, or models and instruments used to confirm the base premise were not always clearly defined or confirmed. Consequently, a more logical approach to a complex human disease like AD is required, and improved collaboration between scientific disciplines is needed to better understand AD pathogenesis and create new and more effective AD treatments. Therefore, large numbers of individuals need to be examined to improve high throughput screening tests for functional metabolic abnormalities in peripheral cells from AD patients.

## CONCLUSION

Despite the continuing dramatic rise in AD frequency, there are no approved pharmaceutical treatments for curing, delaying, or preventing AD. Several studies showed that mitochondrial activity decreases with age and may worsen in the early stages of AD, contributing to its onset. The potential of mitochondria as a target in AD therapy is still being debated, given that certain pharmacological studies were unsuccessful, and while others were promising, none resulted in a marketable AD medication. Nevertheless, current AD knowledge suggests that a comprehensive treatment may remain out of reach for the foreseeable future. However, the enigmatic processes of AD pathobiology also hinder treatment efforts. From this perspective, continued research should be devoted to elucidating the specific AD pathomechanism and investigating prospective AD treatment approaches. Furthermore, clinical evidence is insufficient compared to preclinical data. Consequently, further human studies are required to convert current research findings into clinical use. Mitochondria have a significant role in neurons, maintaining normal neural synapses, signal transduction, and other critical neuronal activities, indicating that targeting mitochondria may be a promising therapeutic option for AD. Understanding AD pathobiology and the pharmacological mechanisms for creating effective treatments may lead to novel neuroprotective AD therapies in the future.

## Figures and Tables

**Fig. (2):**
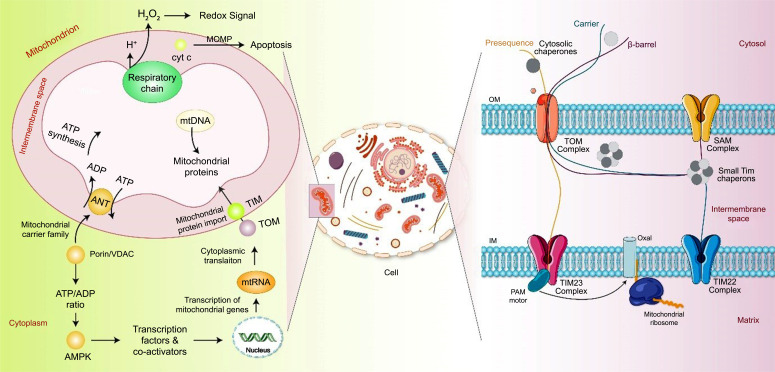
Mitochondrial biogenesis and function. Mitochondria play an important role in ATP synthesis through oxidative phosphorylation. Glycolysis occurs in the cytosol and is responsible for the initial breakdown of glucose into pyruvate. The mtDNA encodes 37 genes involved in synthesizing the respiratory chain and ATP production. Additional proteins are imported into mitochondria by the translocases of the inner (TIM) and outer (TOM) membranes, which are present in the IMM and OMM and are responsible for transporting nuclear-encoded proteins into mitochondria. Additionally, mitochondrial calcium signaling is facilitated by calcium uptake into the mitochondrial matrix by the calcium uniporter (CaU) in response to intracellular calcium fluctuation. Furthermore, mitochondria play an important role in the apoptosis process. When apoptotic signals are received, the OMM is compromised, resulting in OMM permeabilization (MOMP) and the release of cytochrome c (Cyt-c) and other pro-apoptotic proteins from the intermembrane space into the cytosol, causing apoptotic cell death.

**Fig. (3):**
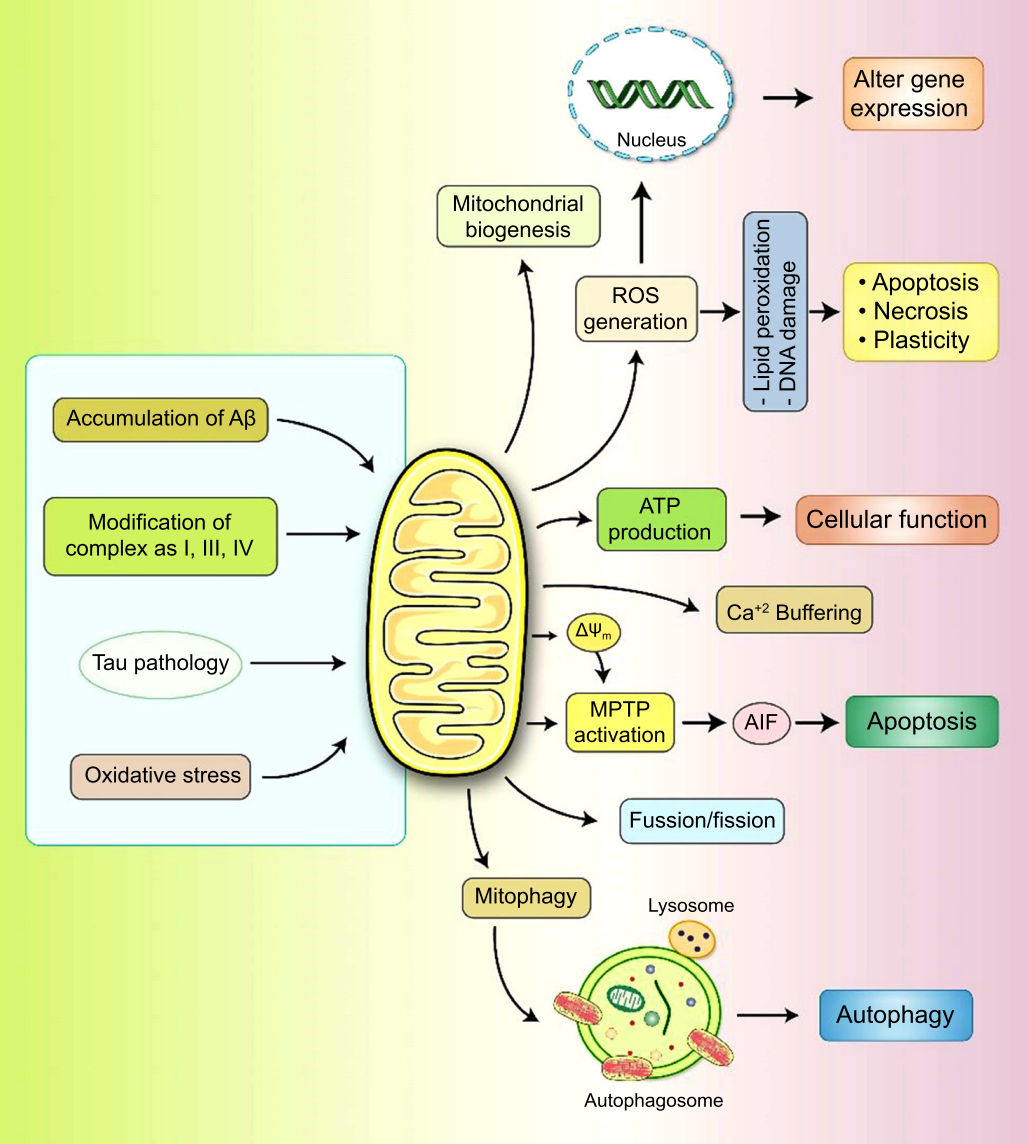
The role of mitochondrial dysfunction in AD etiology. Aβ and tau are known to cause mitochondrial dysfunction, resulting in the modulation of many other variables. ROS production results in the formation of lipid peroxidation and DNA damage, triggering apoptosis. The activation of mitochondrial permeability transition pores (mPTPs) results in damaged mitochondria with decreased ΔΨm, causing the release of Cyt-c and apoptosis-inducing factor (AIF) and the induction of the apoptosis pathway. The antioxidants Aβ and pTau enhance mitochondrial fission and mitophagy.

**Fig. (6):**
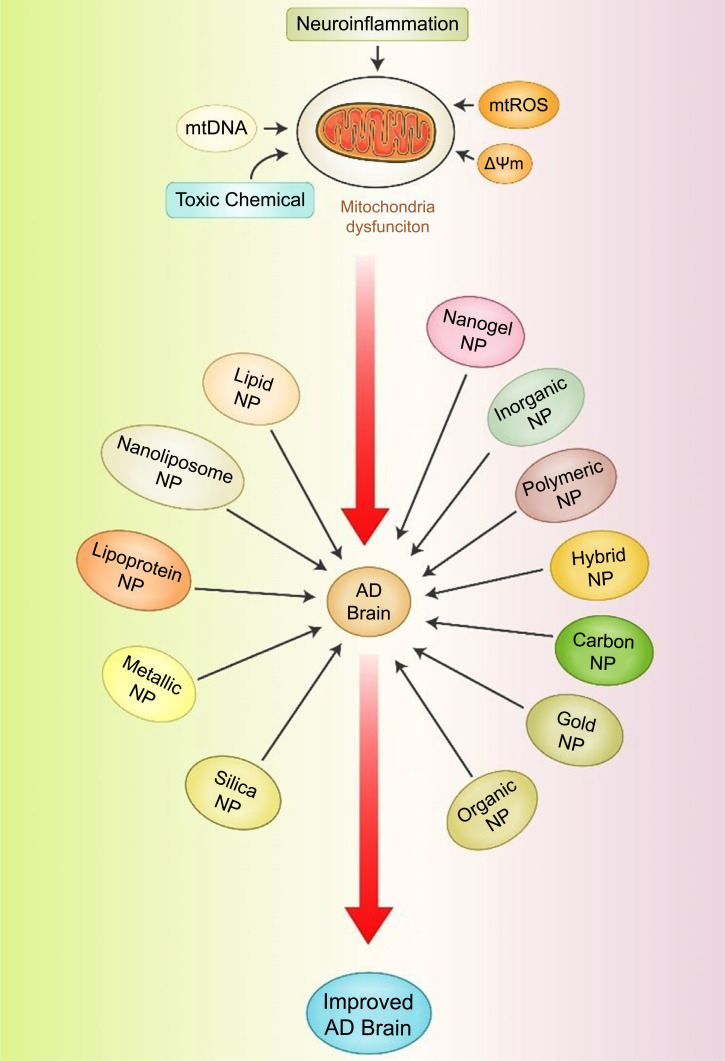
NP-based drug delivery systems for treating AD. NPs can bypass biological barriers, and their use in precision medicine applications may benefit AD treatment. NP designs that increase delivery can potentially improve the performance of precision medicine and the speed at which clinical trials are conducted for AD drugs.

**Table 1:** The effects of several phytochemicals on mitochondrial dysfunctions in AD pathogenesis.

**Phytochemical**	**Experimental Model**	**Pathobiology**	**Molecular Signaling**	**Research Outcomes**	**References**
Liquiritigenin	Aβ-mediated SK-N-MC cell AD model	Mitochondrial fragmentation	MFN1, MFN2, and OPA1 signaling accumulation	Prevent cytotoxicity and mitochondrial fragmentation	[103]
Genistein	APP/PS1 rat model of sporadic AD	Increased Aβ- and tau protein	Autophagy induction and decreased protein aggregates	Enhanced memory and learning function	[101, 104]
Anthocyanins	APPswe double mutation	Oxidative stress and mitochondrial dysfunction	Improved NADH levels	Increased mitochondrial dysfunction	[105]
Quercetin	Sprague-Dawley rat H_2_O_2_-induced neurotoxicity	Oxidative stress	Improved Aβ clearance	Neuroprotection	[106]
Sulfuretin	Aβ neurotoxicity in SH-SY5Y cells and primary hippocampal neurons	Oxidative stress	PI3K/AKT and NRF2/HO activation	Neuroprotection	[107]
Epigallocatechin-3-gallate (EGCG)	Primary cortical rat neurons	Pathological tau species	Improved autophagy and tau clearance	Improved NRF2-dependent tau degradation	[108]
Curcumin	Sprague-Dawley rats	Cerebral ischemia	Improved autophagy by PI3K/AKT/mTOR pathway	Neuroprotection	[109]
Resveratrol	Aβ-induced cytotoxicity in PC12 cells	Oxidative stress	Decreased ROS, activated SOD	Reduced memory impairment and neuroprotection	[110]
Polyphenols	SH-SY5Y neuroblastoma cells	Oxidative stress	Initiation of KEAP1-NRF2 signaling	Neuroprotection	[111]
Kaempferol	Porcine embryos	Oxidative stress	Activated autophagy	Prevented MMP and ROS	[112]

**Table 2:** Nanotechnology-based approaches for the mitochondrial-targeted treatment of AD.

**Drug Candidate**	**Nanoparticle**	**Carrier**	**Model and Effects**	**References**
Anthocyanin	Gold (Au) NPs	Gold colloids	Aβ_1–42_ mouse	[156]
Tanshinone IIA	Cationic bovine serum albumin (BSA)	Polyethylene glycol (PEG)	Rat MCAO and reperfusion	[157]
Berberine	Carbon nanotubes	Phospholipids and polysorbate	Wistar rats in Aβ-induced AD	[158]
Iron oxide	Metallic NPs	Iron crystal functionalized with PEG	*In vitro* amyloid fibrillation experiments	[159]
Curcumin derivative	Liposome	Sphingomyelin	*Ex vivo* CSF model	[160]
Rivastigmine hydrogen tartrate	Mesoporous silica NPs	N-cetyl trimethyl ammonium bromide	Neuroblastoma SH-SY5Y cells	[161]
Galantamine hydrobromide	Solid lipid NPs	Glyceryl behenate lipids	Cognitive deficiency in rats induced with isoproterenol	[162]
Tunable zero-dimension	Carbon dots	Single-walled carbon nanotube	Coarse-grained nanoparticle model	[163]
Angiopep-2-conjugated NPs	Polylactic acid	B6 peptide (transferrin substitute PEG)	Mice injected with aggregated Aβ_1–40_	[164]
Z-DEVD-FMK	Chitosan	Ethylene glycol	Mouse model of middle cerebral artery occlusion (MCAO) and reperfusion	[165]

## LIST OF ABBREVIATIONS

AD: Alzheimer’s Disease
APP: Amyloid Precursor Protein
ATP: Adenosine Triphosphate
BBB: Blood-brain Barrier
CNS: Central Nervous System
ETC: Electron Transport Chain
MMP: Mitochondrial Membrane Potential
mtDNA: Mitochondrial DNA
mtQCS: Mitochondrial Quality Control System
NAD: Nicotinamide Adenine Dinucleotide
NFT: Neurofibrillary Tangle
NPs: Nanoparticles
NRF1: Nuclear Respiratory Factors 1
PD: Parkinson's Disease
PE: Physical Exercise
ROS: Reactive Oxidation Species
SP: Senile Plaques
